# Challenges in Managing Brain Metastases From Merkel Cell Carcinoma in an Immunosuppressed Patient: A Case Report

**DOI:** 10.7759/cureus.98538

**Published:** 2025-12-05

**Authors:** Francisco Rebelo, Teresa Morais Pinheiro, João Pimentel, Carlos Pontinha, Julia Gerhardt

**Affiliations:** 1 Neurosurgery, Unidade Local de Saúde de São José, Lisbon, PRT; 2 Pathology, Unidade Local de Saúde de São José, Lisbon, PRT

**Keywords:** brain parenchyma metastases, immunosuppresion, merkel cell carcinoma (mcc), merkel cell polyomavirus, neuro-oncology surgery

## Abstract

Merkel cell carcinoma (MCC) is a rare but aggressive neuroendocrine tumor with high rates of metastasis and poor prognosis. While MCC commonly metastasizes to the lymphatic system, distant central nervous system (CNS) involvement is unusual, often complicating early detection and treatment. We describe the case of a 58-year-old male with multiple comorbidities who presented with progressive left-sided weakness and disorientation. Imaging revealed a right frontal lesion with mass effect, and further scans identified an iliac and inguinal lymphadenopathy without a primary skin lesion. The initial inguinal lymph node biopsy was inconclusive, prompting surgical resection of the brain lesion. Histopathology confirmed neuroendocrine carcinoma with CK20 dot-like positivity, consistent with MCC. Despite interdisciplinary management, the patient experienced multiple complications, including a respiratory infection and progressive systemic deterioration, ultimately succumbing to septicemia. MCC with CNS metastasis is a rare and highly lethal condition. This case underlines the importance of early recognition and aggressive multidisciplinary management for potential improvement in prognosis, despite limited therapeutic options.

## Introduction

Merkel cell carcinoma (MCC) is a rare and aggressive neuroendocrine tumor of the skin [1,2]. It is considered the most lethal skin cancer, with a five-year disease-specific survival rate of just 64% [2,3]. Although the estimated incidence of MCC is approximately 0.79 new cases per 100,000 people per year, it has tripled over the last two decades, likely due to an aging population, but also to improved diagnostic methods and increased recognition of the disease [2-5].

It is locally aggressive, with a high rate of local recurrence, regional lymph node involvement, and metastasis [3,6]. Between 21% and 50% of patients develop distant metastatic disease [3,7], mainly through the lymphatic system [8-10], with about 50% of the patients having local lymph node involvement at primary diagnosis [9]. Other common sites of distant metastases are the skin, mediastinum, liver, lung, and bone [3].

Central nervous system (CNS) involvement in MCC is rather unusual and may occur via hematogenous spread, lymphatic routes, direct extension, or dissemination through cerebrospinal fluid [3,11]. Due to its rarity, CNS involvement is often detected late, and treatment protocols are not well established [4,8]. Furthermore, brain metastases are associated with a poor prognosis, with a median overall survival of approximately two years without surgical treatment [11].

## Case presentation

A 58-year-old obese (BMI = 35.5 kg/m²), Caucasian male with a medical history of chronic obstructive pulmonary disease (COPD), psoriasis, and type 2 diabetes mellitus, status post renal and pancreatic transplants performed in 2015 for diabetic nephropathy and pancreatic insufficiency, maintained on long-term immunosuppression with tacrolimus, mycophenolate mofetil, and low-dose prednisone, presented to the emergency department with right-sided weakness and two weeks of progressive disorientation. During the physical examination, he exhibited slightly slowed speech, brachial-predominant left hemiparesis with the ability to move against gravity (grade 3/5), and mild left-sided central facial weakness.

**Figure 1:**
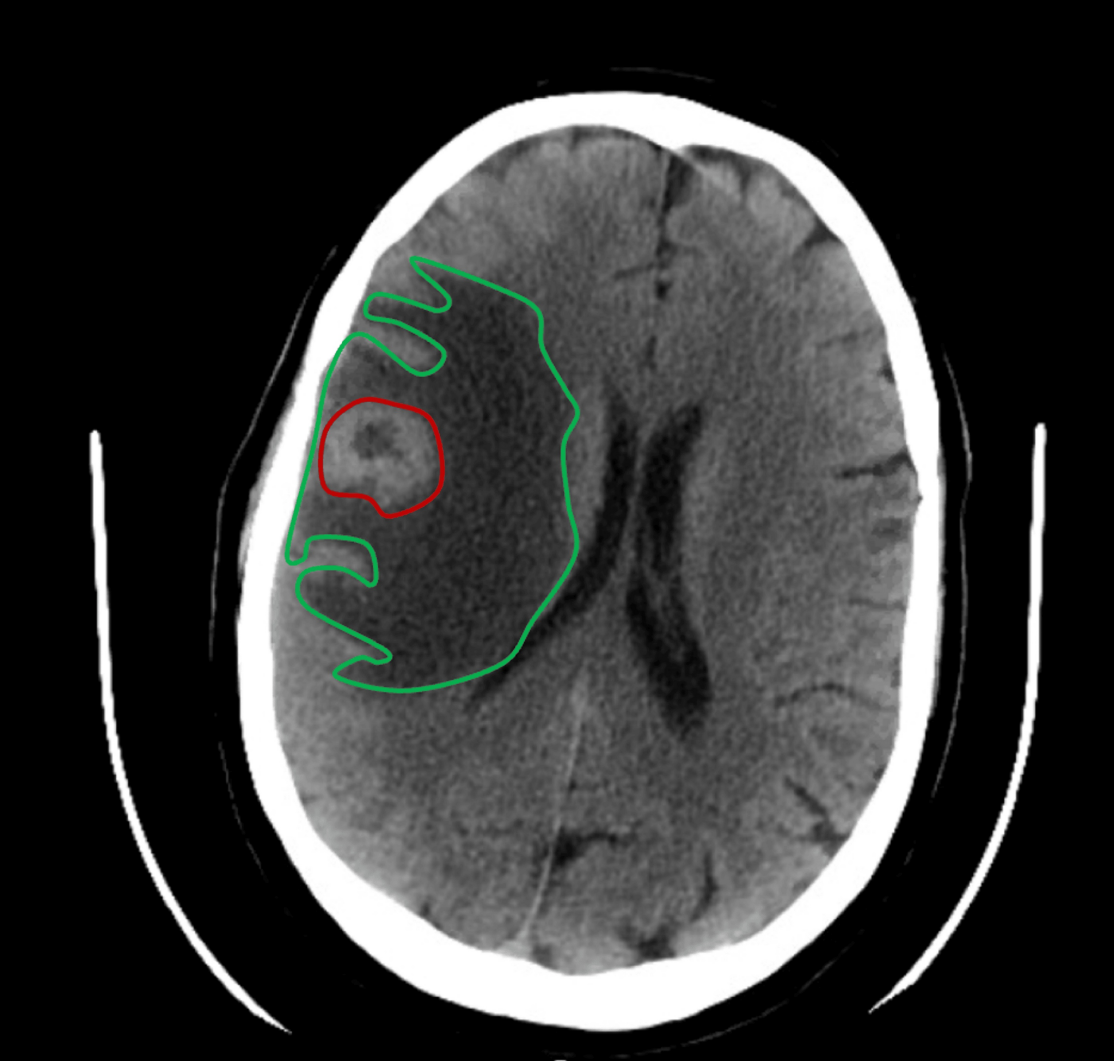
Non-contrast head CT showing a right frontal lesion (outlined in red). A non-contrast computed tomography (CT) scan shows a well-defined, round, space-occupying lesion in the left frontal corticosubcortical region (red circle), measuring 2.6 × 2.7 cm. Associated frontoparietal edema (green circle) extends to the ipsilateral internal and external capsules, causing a 6 mm leftward midline shift.

A non-contrast computed tomography (CT) scan of the head was obtained, revealing a space-occupying lesion with a rounded configuration and relatively well-defined margins at the left frontal corticosubcortical interface, measuring approximately 2.6 x 2.7 cm in maximum anteroposterior and transverse diameters. The lesion was associated with extensive frontoparietal edema, extending to the ipsilateral internal and external capsules, causing a midline shift of approximately 6 mm to the left (Figure 1). 

**Figure 2:**
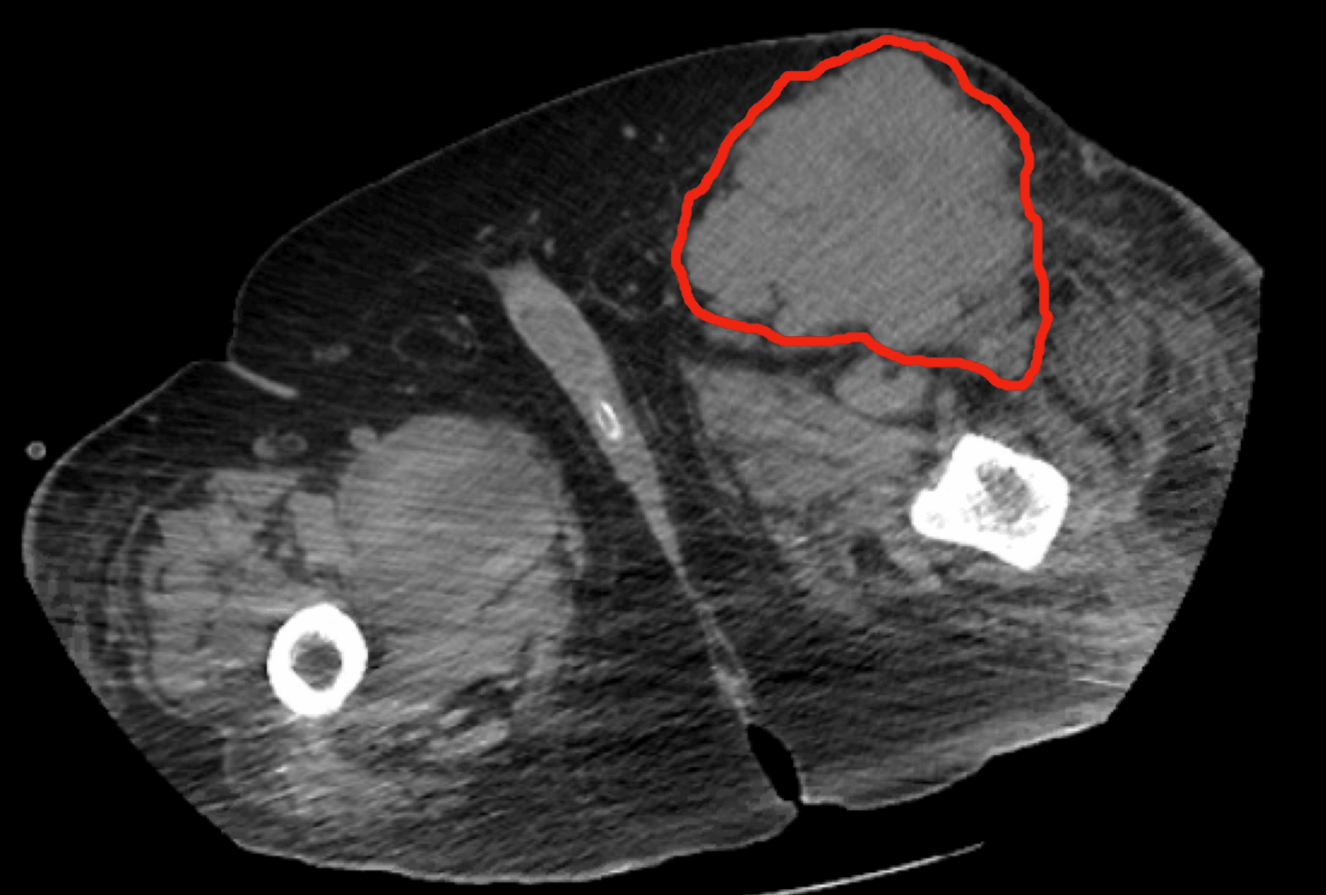
Pelvic CT showing left external iliac and inguinal chain lymph node mass (outlined in red). Pelvic computed tomography (CT) scan showing a lymph node mass involving the left external iliac and ipsilateral inguinal chains (outlined in red), with a maximum diameter of 11 cm. The largest inguinal lymph node demonstrates partial necrosis, raising high suspicion for malignancy.

A thoracic and abdominal CT scan revealed a lymph node mass involving the left external iliac and ipsilateral inguinal chains, with a maximum diameter of 11 cm. The largest inguinal node was partially necrotic, raising high suspicion for malignancy, although no primary tumor was identified (Figure 2). Tumor markers, including Beta-hCG, AFP, CA 19-9, CEA, and PSA, were negative, helping to exclude germ cell, gastrointestinal, and prostate tumors from the initial differential diagnosis based on the patient’s age, sex, and nodal distribution.

Corticosteroid therapy with dexamethasone (4 mg every eight hours) was initiated according to our institutional protocol for patients with symptomatic cerebral metastases and associated edema. At the time of therapy initiation, blood glucose levels were within acceptable ranges for a diabetic patient (fasting: 140 mg/dL; random: 180 mg/dL). The patient was admitted for further investigation. Within the first days of hospitalization, he developed pneumonia, leading to respiratory failure and requiring transfer to the Intensive Care Unit (ICU). The patient’s condition then deteriorated, progressing to septic shock and requiring vasopressor support. Once stabilized and showing gradual clinical improvement, he underwent a brain MRI that revealed a roughly rounded intra-axial mass in the right frontal cortico-subcortical region. The lesion exhibited intermediate signal intensity on T1 (mostly hypointense) and T2 (mostly hyperintense), with diffusion restriction (mean ADC ~0.9 x 10¯³mm²/s) and extensive surrounding vasogenic edema, causing mild mass effect and a slight leftward shift of the midline structures (about 5 mm). 

**Figure 3:**
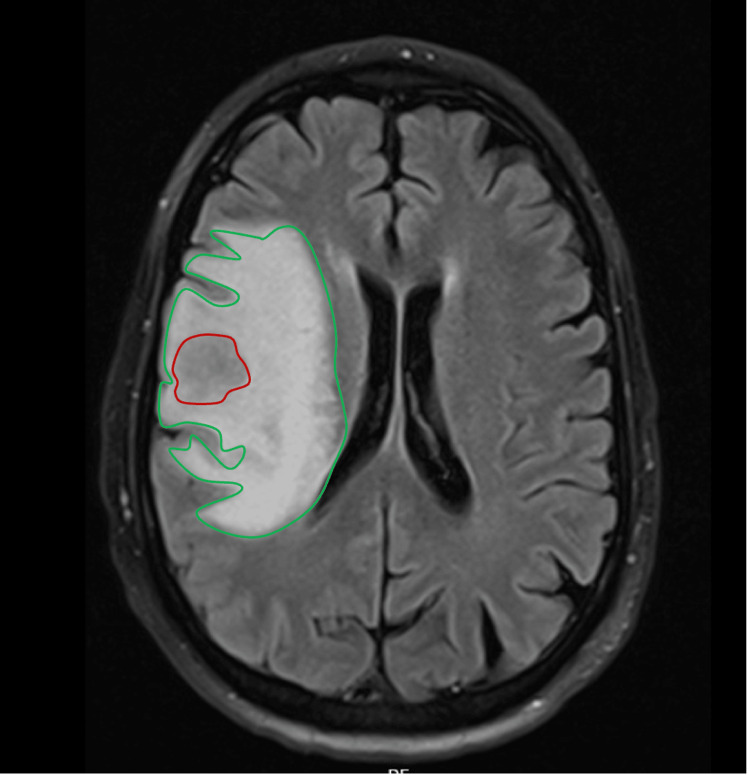
MRI showing a right frontal (outlined in red) mass surrounded by extensive vasogenic edema (outlined in green). Magnetic resonance imaging (MRI) of a right frontal cortico-subcortical mass is shown in a panel of four images: (a) T2 fluid-attenuated inversion recovery (FLAIR) image, showing the lesion (red circle) surrounded by extensive vasogenic edema (green circle).

**Figure 4:**
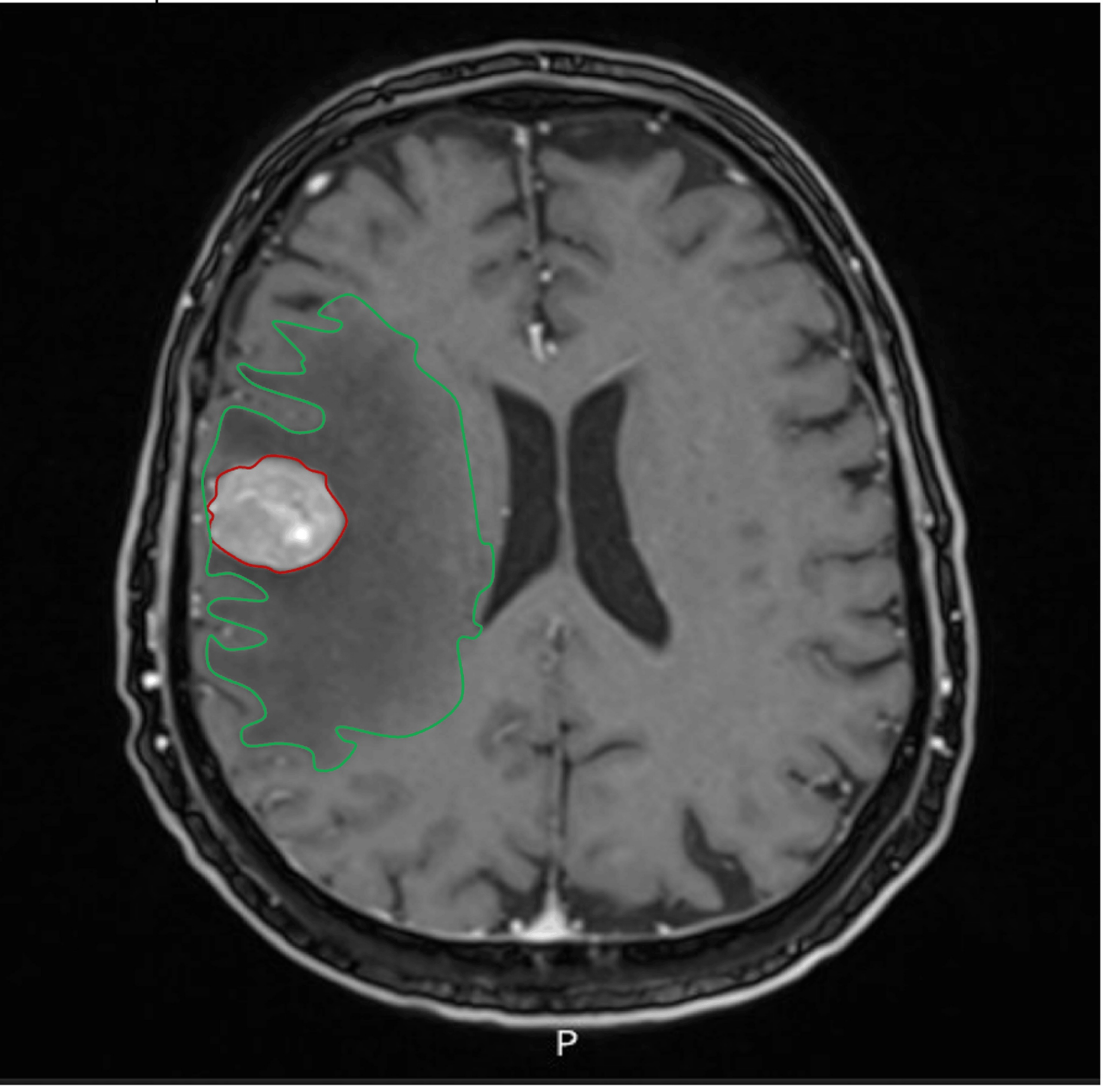
Post-gadolinium T1-weighted MRI image showing a heterogeneous enhancement. Post-gadolinium T1-weighted magnetic resonance imaging (MRI) image showing intense, nodular, and somewhat heterogeneous enhancement, particularly in the central part of the lesion, with a small non-enhancing area. The lesion (red circle) and the surrounding vasogenic edema (green circle) are indicated.

After gadolinium administration, there was intense, nodular, and somewhat heterogeneous enhancement, particularly in the central part of the lesion, with a small non-enhancing area. The lesion measured 34 × 36 × 34 mm on MRI, compared with 26 × 27 mm on the prior CT scan, reflecting improved delineation on MRI (Figure 4).

**Figure 5:**
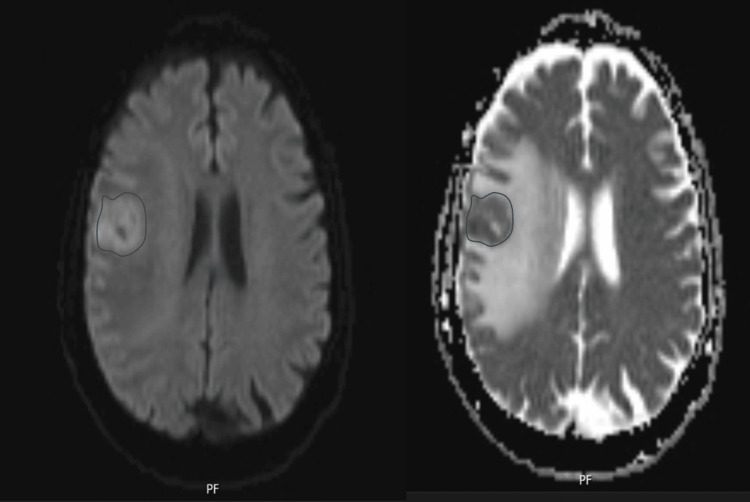
Diffusion-weighted imaging (DWI) MRI demonstrating restricted diffusion. Diffusion-weighted imaging (DWI) Mmagnetic resonance imaging (MRI) demonstrating restricted diffusion in a right frontal cortico-subcortical lesion measuring 34 × 36 × 34 mm. The area of diffusion restriction is indicated by a blue circle. The mean ADC value is approximately 0.9 × 10⁻³ mm²/s, indicating low diffusion.

Diffusion-weighted imaging (DWI) demonstrated restricted diffusion with a mean ADC value of approximately 0.9 x 10^-3^ mm/s (Figure 5).

Spectroscopy showed a significant decrease in N-acetylaspartate (NAA), increased Cho, with an inverted Cho/Cr ratio, NAA/Cho > 2, and a Lac/Lip peak (Figure 3). 

**Figure 6:**
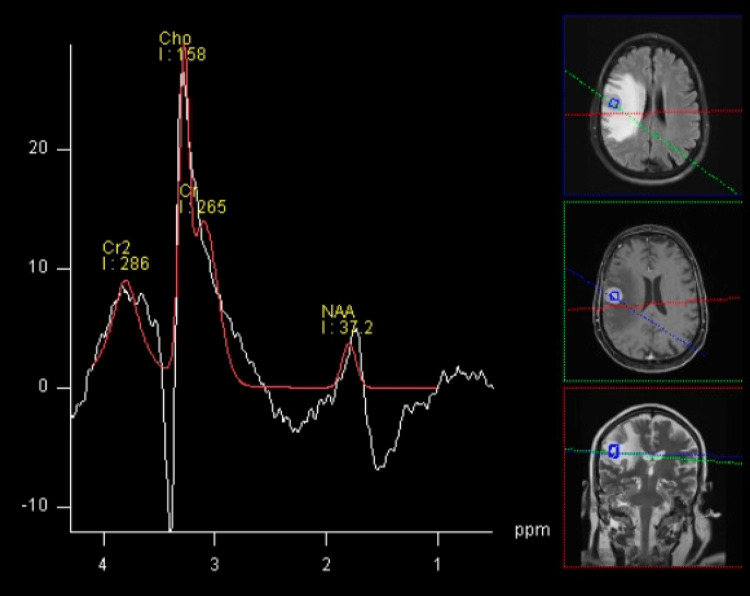
MRI spectroscopy. Spectroscopy showed a significant decrease in N-acetylaspartate (NAA, marker of neuronal integrity), increased choline (Cho, reflecting cellular turnover and membrane synthesis), with an inverted Cho/Cr ratio, NAA/Cho > 2, and a lactate/lipid (Lac/Lip) peak. These findings indicate a high-cellularity malignant lesion, with neuronal loss, increased membrane turnover, and areas of anaerobic metabolism.

The General Surgery team performed a biopsy of the most prominent inguinal lymph node, but the results were inconclusive due to the presence of non-viable cells.

Differential diagnosis and treatment

The patient was evaluated by an interdisciplinary team. Following recovery from pneumonia, persistent neurological symptoms caused by the mass effect of the right frontal lesion, together with its size and uncertain etiology, prompted consideration for surgical intervention.

Given the patient’s age, sex, comorbidities, and immunosuppressed status, as well as the presence of large metastatic lymph nodes in the left external iliac and ipsilateral inguinal regions, the initial differential diagnosis focused primarily on metastatic carcinoma. In adult males, lung and prostate cancers are among the most frequent malignancies and were initially considered; however, thoracic CT scans revealed no evidence of a primary tumor, and PSA levels were normal, making these diagnoses less likely. The inguinal lymph node involvement raised suspicion for a cutaneous primary malignancy or other neuroendocrine tumors capable of distant metastasis. In addition, given the patient’s immunosuppression, atypical malignancies such as post-transplant lymphoproliferative disorder (PTLD) or other hematologic neoplasms were considered. Tumor markers, including Beta-hCG, AFP, CA 19-9, CEA, and PSA, were negative, helping to exclude germ cell, gastrointestinal, and prostate tumors from the differential diagnosis.

**Figure 7:**
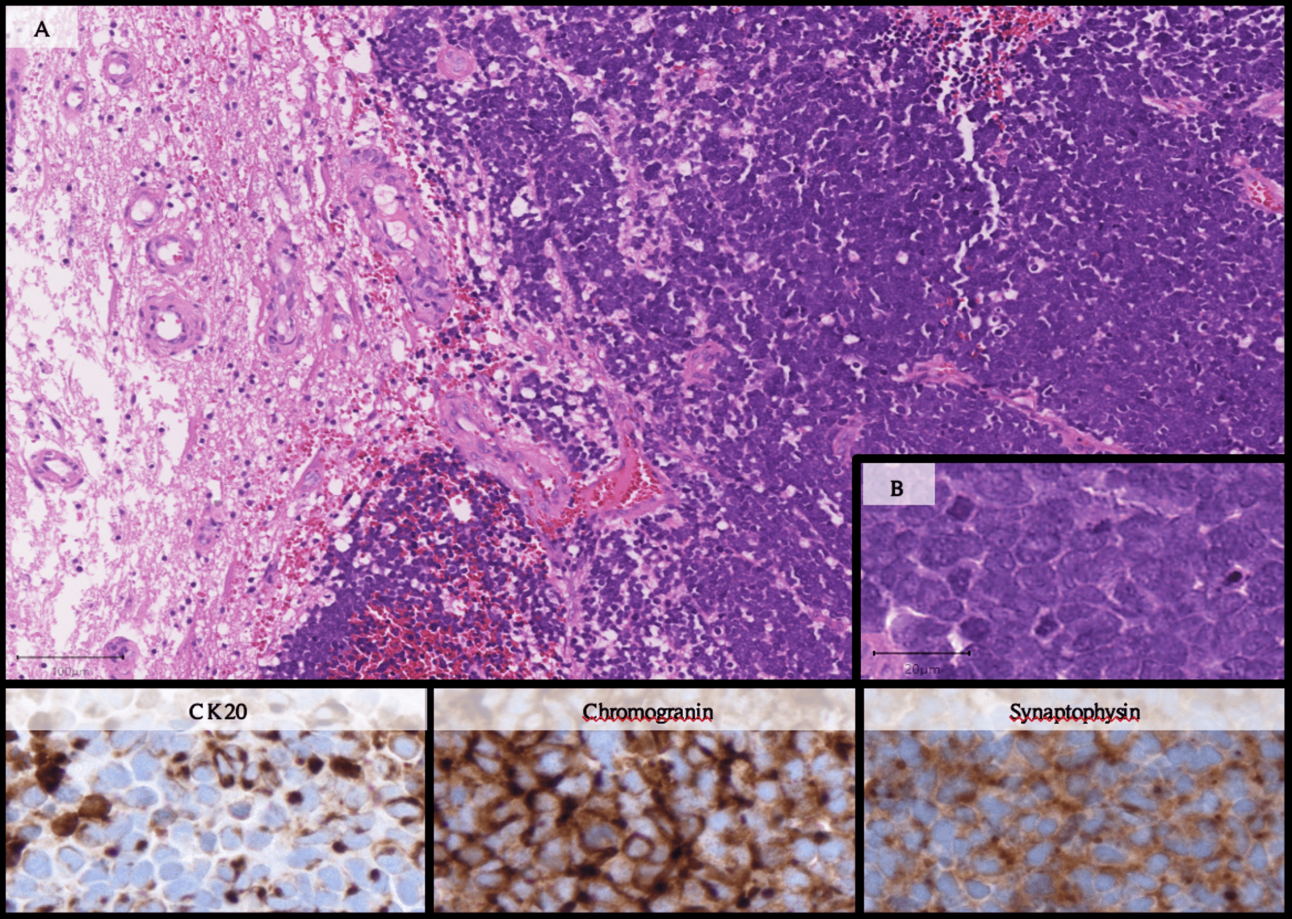
Microscopic examination of Merkel cell carcinoma. Histopathological analysis revealed a malignant epithelial neoplasm with solid growth and abundant necrosis (A). The neoplastic cells were of intermediate size, with scant cytoplasm and hyperchromatic nuclei featuring a "salt and pepper" chromatin pattern (B). The cells were positive for CK20 (dot-like paranuclear pattern) (lower left image), chromogranin (lower middle image), and synaptophysin (lower right image), and negative for TTF1, p40, CD45, and GFAP, among other markers. These findings confirmed the diagnosis of Merkel cell carcinoma (MCC) metastasis.

A right frontal craniotomy was performed, and gross total resection of the lesion was achieved based on intraoperative assessment and later confirmed by postoperative MRI; the procedure was uneventful. Concurrently, a new inguinal lymph node biopsy was obtained. Histopathological analysis of the cerebral lesion (Figure 7) revealed a metastasis of a neuroendocrine neoplasm with partial necrosis. The neoplastic cells expressed cytokeratins (AE1/AE3, CAM5.2, CK20), chromogranin A, and synaptophysin, and were negative for CK7, S100, PSA, and GATA3. The tumor proliferation index (Ki-67) was approximately 40%, and TTF-1 staining was negative. The dot-like expression of CK20 supported the final diagnosis of MCC.

Outcome and follow-up

Initial postoperative cranial CT revealed no significant changes in the surrounding edema, but inflammatory markers improved shortly after. However, the patient then developed an infection at the central venous catheter (CVC) site, leading to fever, tachypnea, and tachycardia. He was transferred to the ICU, where empirical antibiotics and vasopressor support were initiated. MRSA was isolated from the infection site, prompting an adjustment in the antibiotic therapy. Given the histopathological findings, dermatology and oncology consultations were requested. Dermatology assessed the patient and found no skin lesions indicative of primary cutaneous MCC, and oncological evaluation for targeted therapy remained pending due to the patient’s poor Karnofsky performance status. Later, the patient developed wound dehiscence and a left inguinal abscess, addressed by the general surgery team. Despite these interventions, the patient’s condition continued to deteriorate due to the rapid progression of MCC and complications from prolonged hospitalization, including multiple infections. Septicemia from the CVC-associated infection, with suspected endocarditis, led to a shift toward comfort care. The patient passed away approximately 2.5 months after initial diagnosis, reflecting the very rapid progression and aggressive nature of the disease.

## Discussion

MCC is a rare neuroendocrine tumor characterized by small round cells, with a monomorphic appearance, minimal cytoplasm, high mitotic index, and frequent apoptotic bodies [7]. The exact origin of MCC is uncertain, but it is thought to derive from Merkel cells, which are mechanoreceptors that detect light touch but also have neuroendocrine and immune functions [5,8]. These cells were first identified by anatomist Friedrich Sigmund Merkel in 1875 [12]. Diagnosis was historically challenging due to its varied histology, but has become more reliable nowadays with the advances in immunohistochemistry, particularly cytokeratin-20 (CK-20) staining [2,13]. CK-20 is positive in approximately 87% of cases and helps distinguish MCC from small cell lung cancer, which is typically CK-20 negative [7,14]. Other markers, such as A-chromogranin, enolase, and CD56, have lower sensitivity, with positivity rates of 52%, 50%, and varying percentages, respectively [7]. In this report, diagnosis was confirmed by dot-like CK-20 expression, along with A-chromogranin and synaptophysin positivity. The absence of CK7, TTF-1, and GATA3 helped exclude other neuroendocrine malignancies. 

Merkel cell polyomavirus (MCPyV), discovered in 2008, is present in ~80% of MCC cases, while UV-induced mutations drive MCPyV-negative tumors, indicating distinct oncogenic pathways [9,13,15-18]. Immunosuppression significantly increases MCC incidence, particularly in transplant recipients, B-cell malignancies, and HIV-positive patients [19,20]. By impairing immune surveillance, it facilitates MCC development [20]. In this case, the patient’s history of renal and pancreatic transplants and chronic immunosuppression likely contributed to the development of MCC. In Portugal, routine testing for this virus is not widely available, and systematic screening is not performed, despite its crucial diagnostic and prognostic implications. Studies indicate that MCV-positive patients have better survival outcomes, underscoring the importance of identifying viral status.

MCC remains rare, with about 2,500 new cases annually in the EU, leading to roughly 40% of cases resulting in death [16]. However, its incidence has risen significantly in recent years; for instance, Hodgson et al. reported a rise from 0.15 to 0.79 cases per 100,000 people, with males affected almost three times more than females [15,20,21]. This increase is attributed to an aging population, more UV exposure, and better diagnostic methods [15]. MCC most commonly affects elderly Caucasian men, with an average diagnosis age of 69-70 years, and is often found on sun-exposed areas, frequently alongside other UV-related skin cancers [4,6,8,11]. MCC has a strong tendency for local recurrence and lymphatic spread, with about 50% of patients developing metastatic disease [14,19]. It typically presents as painless, erythematous to violaceous nodules on sun-exposed areas, which may ulcerate and grow rapidly [3]. The typical presentation is summarized by the acronym AEIOU: asymptomatic, rapidly expanding, occurring in immunosuppressed individuals over 50, and found on UV-exposed fair skin, with 89% of patients displaying at least three of these features [9,13]. Around 50% of patients have lymph node involvement at diagnosis, and in 4-5% of cases, the primary tumor cannot be identified, which is typically linked to a more favorable prognosis [8,9,21,22]. In our case, while lymph node involvement was present, the patient's immunosuppression likely contributed to a worse outcome.

Brain metastases from MCC are rare, but their incidence seems to be increasing due to improved survival rates and advances in diagnostic imaging [14,23]. Since 1982, reports of neurometastatic MCC have steadily increased, with 40 cases described to date [8,24]. MCC typically spreads hematogenously to the CNS, although it can also extend directly or through cerebrospinal fluid (CSF) [24]. Diagnosis is challenging as the primary tumor may not always be identifiable, and symptoms can be subtle [11,22]. While bone destruction is uncommon, small emissary veins may facilitate intracranial spread [3,24]. Perineural spread to the brain, including leptomeningeal dissemination, has been reported in rare cases of MCC [12]. Patients with metastatic disease at diagnosis may have distinct tumor biology, and the improved prognosis observed after lymph node removal may relate more to tumor characteristics than treatment [9].

MCC treatment remains complex, as no established guidelines exist [9,25]. Given the rarity of brain metastases, clinical approaches vary across institutions [23]. Management is typically multimodal, with no universally accepted regimen [22,26]. While surgical resection is commonly recommended for primary MCC, there is less standardization when it comes to managing brain metastases, and current literature offers limited guidance [2,7]. Surgical excision is considered the first-line approach for accessible, symptomatic brain metastases, aiming to alleviate neurological symptoms and reduce recurrence risk [22,26]. A pooled analysis of 40 patients with MCC brain metastases revealed that treatment often involves surgery, radiotherapy (RT), and systemic therapies [1,2]. Radiotherapy has yielded favorable results in some cases, although its precise role in treatment remains somewhat limited [22,26]. Stereotactic radiosurgery (SRS), a key modality in treating brain metastases from MCC, has demonstrated effectiveness, likely due to MCC's radiosensitivity [2,26]. When combined with resection, SRS or RT tends to improve survival rates, although more research is necessary to determine the optimal sequence of these treatments [21]. Chemotherapy, however, has proven less effective in managing brain metastases from MCC, although it may still be useful in specific cases [3]. Immunotherapy with PD-L1/PD-1 monoclonal antibodies (e.g., pembrolizumab, nivolumab, avelumab) is being used in advanced MCC and has been showing a promising potential for long-term disease control and improved survival. However, few cases of MCC brain metastasis treated with immunotherapy have been reported [12,21]. Some studies suggested that the combination of immunotherapy with surgery and SRS could enhance outcomes. Grubb et al. reported the use of adjunct pembrolizumab and SRS after resection of a right MCC brain metastasis in a patient, with stable disease at the six-month follow-up [2,17]. In our case, surgical resection was prioritized over systemic therapy due to the patient’s neurological deterioration and the need for a definitive diagnosis. Imaging was inconclusive, and the initial biopsy was non-diagnostic, delaying treatment. Given the lesion’s size and persistent deficits, resection provided both symptom relief and histopathological confirmation. In addition, his poor performance status, immunosuppression, and recurrent infections made systemic therapy unfeasible, ultimately leading to a shift toward supportive care.

The prognosis for patients with brain metastases from MCC is generally poor, with most cases managed palliatively due to limited treatment options and low survival rates in advanced stages, staying relatively unchanged over time [13,22]. A study by Yi-Jun Xia et al. found that the five-year survival rate for patients with distant metastases is around 18%, and while treatments like lymph node surgery, radiotherapy, and chemotherapy may help, their impact is limited for advanced disease [9]. MCC prognosis is notably worse than melanoma, with five-year survival rates for MCC around 41%, compared to 93% for melanoma in a Queensland cohort [27]. Other factors, such as immune status and the characteristics of the primary tumor, also affect survival. Patients with nodal MCC of unknown primary origin tend to have nearly double the survival rates of those with identifiable primary lesions, possibly due to stronger immune responses and higher tumor mutational burdens [27]. The median survival after brain metastasis from MCC ranges from 12 to 60 months, highlighting the aggressive nature of the cancer once it spreads to the CNS [1].

## Conclusions

We describe a case of a male patient with brain metastasis as the first manifestation of MCC. This case illustrates the clinical challenges of managing MCC, including its rapid progression and complex treatment. It emphasizes the importance of early detection and effective treatment strategies. The patient’s comorbidities, advanced stage at presentation, and the lack of targeted therapies for brain metastases contributed to the unfavorable outcome. While there are no formal treatment guidelines, emerging evidence suggests that a multimodal approach involving surgical resection, radiotherapy (particularly SRS), and possibly immunotherapy may offer the best chance for disease control. This underscores the need for further research into effective treatments, particularly for CNS involvement, and the importance of early detection to improve prognosis. Future research should focus on refining treatment protocols and exploring new therapeutic options to enhance survival and quality of life for patients with advanced MCC.
